# Medical Missions

**Published:** 1898-08-27

**Authors:** 


### The Hospital

A Journal of

The Medical Sciences and Hospital Administration.

Vol. XXIV.—No. 622.] Edited by Sir Henry Burdett, K.C.B., and Solomon C. Smith, M.D., M.R.C.P. [Aug. 27, 1898.

### Medical Missions.

The publication by Sir Henry Acland, with appended notes and correspondence, of an address which he delivered in December to the Oxford University Junior Scientific Club, on the subject of Medical Missions in their Relation to the University, calls attention, in scholarly and thoughtful fashion, to a question which recent events in India, not to speak of other countries, have brought into considerable prominence. It is neither more nor less than to determine the part, if any, which the medical profession in general, and the medical graduates of Oxford in particular, may fruitfully take in the work of disseminating a knowledge of Christianity among heathen races, using the term to include not only the comparatively uncivilised, but also the adherents of such religions as Brahminism, Buddhism, and Islamism. Sir Henry prefaces his book by an additional note, taken from the *Spectator* of June 25th, in which that journal expresses a belief that the resistance to modern sanitation offered by the mobs of Bombay and other Indian cities must be taken as evidence of a necessity to abandon the policy of which the "Plague Rules" were an expression, and to wait for any further advance in this direction until the knowledge already possessed by native doctors, educated on European lines, has filtered down into the community more deeply than at present, even though the consequence of waiting may be the needless loss of many thousands of lives. We cannot but hasten to express our entire dissent from so dangerous a doctrine. The business of the medical profession is everywhere to save lives, even, if necessary, by the enforcement of precautions distasteful to those upon whom they are imposed; and, with all due respect for our contemporary, our motto at least must be "*Fiat justitia, ruat cœlum.*"

The immediate cause of the delivery of Sir Henry's address was the receipt of a letter from Sir Thomas Grainger Stewart, calling his attention, as the chief medical authority of Oxford, to the Medical Missionary Society which has long existed at Edinburgh, and inviting Oxford men to cast in their lot with it and to become partakers in its work. Sir Henry meets the invitation with warm sympathy as to its objects, and with an entire conviction that it is the duty of higher races to act as missionaries to the lower, and to strive for their physical as well as for their spiritual or mental improvement; but he evidently feels some doubt as to the desirableness of combining the medical and the missionary character in a single individual, while at the same time he declares that no mission can be perfectly equipped unless it numbers a medical man among its members. We feel that in thus advising the combination of the two callings, but the performance of the work by different hands, Sir Henry is speaking with the sagacity of which he has given so many previous examples, and is giving the counsel best calculated to promote at once the welfare of missions and the advancement of Christianity.

There can be very little doubt that the spread of Christianity over the world has been greatly hindered in past times by the fact that too many missionaries have been ignorant of most things outside the boundaries of dogmatic theology, and have failed to realise the extreme difficulty of transplanting a system of religion, itself of gradual growth, under a given set of conditions, into conditions of a wholly alien character, and through the medium of an imperfectly understood language, or, in many cases, of a native language possessing no words corresponding to the ideas which the missionaries have desired to convey. As regards both of these difficulties, the presence of a medical colleague, trained to observe the operations of the human mind, and to separate their essence from their symbols, could hardly fail to be of enormous practical value; while, at the same time, his ministrations within his own sphere would display an evidence of goodwill to the lower race, and would be attended by such continuous and undeniable benefit to its members, as could hardly fail to secure their approval, perhaps even their gratitude, so far as that might be dependent upon a sense of favours to come, and, on the whole, to induce them to turn willing ears to the teaching of the clerical members of the body with which the doctor was associated. Such a position would hardly be sought, nor could it with any fitness be filled by any but a religious person, devoted to his work both for its own sake and in so far as it was an accessory to Christian teaching, and prepared to show by his daily life an example of the virtues which Christianity inculcates, and which all men, of whatever creed, can understand and appreciate. Beyond this we should not be prepared to go. No man can serve two masters, or can excel in two such very different departments of duty as the work of healing the sick and that of evangelising the heathen, both of which are too engrossing to be performed by turns, as opportunities may serve, and our own conviction is that the assistance given to missions by medicine may be properly limited to the rendering of medical services, but to rendering them in such a manner as to bring them into complete harmony with the general purposes of the establishment of which the doctor is a member.

